# Nanoscale α Phase Enables Excellent Strength–Ductility Balance in TC21 Titanium Alloy

**DOI:** 10.3390/nano16070442

**Published:** 2026-04-05

**Authors:** Keyu Ma, Zehua Jiang, Kaihong Wu, Yongfeng Shen, Zhaodong Wang

**Affiliations:** 1 Key Laboratory for Anisotropy and Texture of Materials (Ministry of Education), School of Materials Science and Engineering, Northeastern University, Shenyang 110819, China; 2 State Key Laboratory of Digital Steel, School of Materials Science and Engineering, Northeastern University, Shenyang 110819, China

**Keywords:** TC21 alloy, nanoscale α phase, mechanical properties, strengthening mechanism, ductility

## Abstract

The limited ductility of conventional titanium alloys significantly limits their application in critical load-bearing components. To overcome this limitation, a Ti-6Al-2Mo-2Nb-2Zr-2Sn titanium alloy (TC21) was subjected to warm rolling at 500 and 600 °C and aging treatment. Subsequently, microstructural characterization was conducted using scanning electron microscopy, electron backscatter diffraction and transmission electron microscopy, while the mechanical properties were tested by uniaxial tensile tests and nanoindentation tests. The sample warm rolled at 600 °C exhibited an optimal combination of strength and ductility, with an ultrahigh yield strength of 1138 MPa and an elongation-to-fracture of 7.3%. Aging treatment further enhanced the yield strength to 1263 MPa, while retaining a good ductility of 9.6%. The improved mechanical properties are mainly associated with the formation of nanoscale secondary α phase (α_s_) lamellae caused by the aging treatment. Interface strengthening is identified as the primary strengthening mechanism. In particular, the optimal volume fraction and decreasing texture intensity of the soft phase contribute to the enhanced ductility. This work provides a method for viable thermo-mechanical processing for achieving an excellent strength–ductility combination in titanium alloys.

## 1. Introduction

Titanium alloys are extensively utilized in high-tech industries, particularly aerospace, owing to their exceptional mechanical properties, low density, and excellent heat resistance [1,2,3,4]. However, conventional titanium alloys often exhibit limited ductility, which restricts their potential application in critical load-bearing components [5]. Consequently, enhancing ductility without compromising strength remains a challenge. This strength–ductility trade-off represents a fundamental dilemma in materials science and has motivated extensive research over the past decades.

Substantial efforts have been devoted to improving the mechanical properties of titanium alloys through microstructural control. Commercial titanium alloys are generally categorized into α, β, and α + β types [6]. While α titanium alloys offer superior thermal stability and corrosion resistance, their strength and elastic modulus are relatively low [7]. β titanium alloys demonstrate high strength but suffer from inadequate high-temperature performance [8]. In contrast, α + β titanium alloys have garnered significant attention due to their excellent strength–ductility balance, fatigue resistance, and better creep and heat resistance compared to β alloys. The outstanding mechanical properties of α + β titanium alloys originate from complex interactions between α and β phases and their intricate microstructures [9,10]. The strength differential between these phases can induce hetero-deformation-induced (HDI) hardening near the α/β phase boundaries. When subjected to mechanical loading, the strength mismatch between phases creates geometrically necessary dislocations (GNDs) that, when accumulated at phase boundaries, generate long-range back stresses that enhance work hardening and delay necking [11,12]. Zhang et al. [13] revealed that coherent α/β interfaces significantly enhance both strength and toughness in titanium alloys, facilitated by nano martensite–dislocation interactions. Similarly, the work of Dumas et al. [14] on a Ti-4.5Al-2.5Fe-0.25Si alloy demonstrated outstanding work-hardening capability and high yield strength, attributable to its α + α’ + β multiphase microstructure. In recent years, the design of heterostructure materials, which consist of domains with dramatic strength differences, has emerged as a prominent strategy for overcoming the strength–ductility trade-off.

The mechanical behavior of titanium alloys is highly sensitive to temperature, cooling rate, and strain rate due to their complex microstructures [15,16]. This sensitivity implies that microstructural evolution—and hence, mechanical properties—can be tailored through heat-treatment design. For instance, increasing the annealing temperature was found by Chen et al. [17] to promote the α → β-phase transformation, accelerate static recrystallization (SRX), and stimulate grain growth. Further extending this concept, a tri-modal microstructure consisting of equiaxed α, lamellar α, and transformed β phases was developed by Zhang et al. [18] through dual- or triple-heat treatments, leading to enhanced tensile properties in TA15 (Ti-6.5Al–2Zr–1Mo–1V) alloy. Shao et al. [19] revealed that the thickness of the lamellar α phase is a critical parameter governing the yield strength of titanium alloys. Gao et al. [20] indicated that variations in β-phase morphology lead to different deformation mechanisms, improving both toughness and yield strength. Furthermore, grain size can be optimized by adjusting heat-treatment temperatures to enhance performance. By tailoring the β-phase grain size in Ti-1023 (Ti-10V-2Fe-3Al) alloy, Li et al. [21] effectively enhanced the yield strength, increased the critical stress for β-phase transformation, and consequently delayed the onset of the transformation process. Therefore, optimizing the heat-treatment process is crucial for enhancing the performance of titanium alloys. Particularly, the precise control of α-phase precipitation—including its morphology (lamellar or equiaxed), size, and volume fraction—during heat-treatment process is pivotal for tailoring the mechanical response. Controlling the rolling temperature can regulate the precipitation of α and β phases and inhibit the growth of excessively coarse grains, thereby improving overall comprehensive performance. The ideal microstructure should therefore comprise dual-scale architecture: coarse phases that accommodate plastic deformation and nanoscale precipitates that provide effective barriers to dislocation motion. Such heterogeneous structures have recently emerged as a new strategy to overcome the strength–ductility trade-off in various material systems.

Due to poor ductility and high deformation resistance, cold rolling at room temperature often induces cracking in titanium alloys [22]. Low processing temperatures can also reduce work hardening, lead to insufficient recrystallization, and result in an inhomogeneous microstructure [23]. Consequently, warm rolling and hot rolling are commonly employed for shaping titanium alloys. Hot rolling refers to rolling conducted above the recrystallization temperature, whereas warm rolling is carried out below the recrystallization temperature but well above room temperature. Warm rolling can enhance fatigue life and durability by mitigating void formation [24,25]. It also requires lower temperatures and energy consumption compared to conventional heat treatments, presenting an attractive strategy for energy conservation. Furthermore, warm rolling does not lead to a severe degradation of the alloy’s performance. Thus, applying warm rolling processes to titanium alloys is both technologically meaningful and beneficial for formation and deformation control.

In this study, a novel thermo-mechanical processing route combining warm rolling with subsequent aging is designed for the Ti-6Al-2Mo-2Nb-2Zr-2Sn (TC21 designated according to GB/T 3620.1-2016 [26] alloy. The aim is to obtain a well-balanced microstructure with an excellent combination of strength and ductility. Furthermore, this study seeks to provide a deeper understanding of the deformation mechanisms, particularly the synergy between different secondary α-phase (α_s_) morphologies and the role of α_s_/β interfaces under loading. The results indicate that the alloy maintains a relatively high yield strength of 1263 ± 10 MPa with an elongation-to-fracture of 9.6%. The present work aims not only to optimize the strength–ductility balance but also to quantitatively decouple the underlying strengthening mechanisms through a combined experimental and theoretical approach. Quantitative analysis reveals that the α/β interface contributes 67% of the overall strength, serving as the primary strengthening source. This comprehensive understanding is expected to provide a more general guideline for the thermo-mechanical processing of dual-phase titanium alloys.

## 2. Materials and Methods

### 2.1. Material Preparation

The TC21 titanium alloy in this study was provided by the Northwest Institute of Nonferrous Metal Research of China (Xi’an, China). The nominal chemical composition of the alloy is Ti-6Al-2Mo-2Nb-2Zr-2Sn-1Cr. In the forged alloy, the α phase was uniformly distributed in the matrix of the β-transformation structure, forming a characteristic equiaxed microstructure. The real chemical compositions determined through chemical analysis are listed in Table 1, which meets the requirements of the GB/T 3620.1-2016. Using a DIL-805A/D (Waters Technology, Shanghai, China) dilatometer, the α + β→β transformation temperature (T_β_) of the alloy was determined to be approximately 965 ± 5 °C.

**Figure 1:**
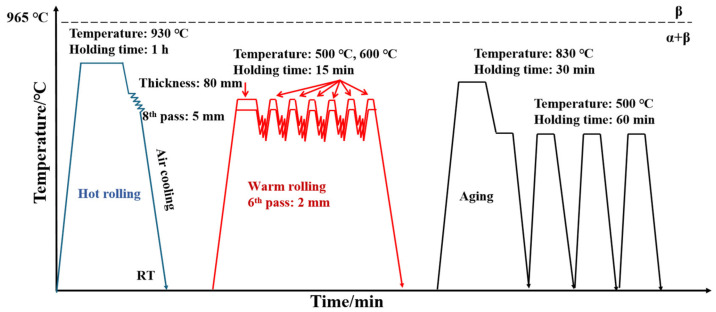
Heat-treatment processing of TC21 alloy.

Specimens with dimensions of 100 mm × 80 mm × 80 mm were cut from the received TC21 alloy and then were subjected to heat treatment at 930 °C for 1 h followed by hot rolling on a Φ 450 mm × 400 mm two-high reversible high-strength hot-rolling mill. The alloy was hot rolled from an initial thickness of 80 mm to 5 mm in 8 passes, achieving a total reduction of 93%. The alloy was subsequently air cooled to room temperature. Subsequently, a billet with dimensions of 80 × 60 × 5 mm^3^ was cut along the rolling direction for warm rolling. The samples were held at 500 °C and 600 °C for 15 min and then warm rolled, with a total reduction of 60% after 6 passes. Subsequently, the samples were air cooled to room temperature (RT). The specimens are referred to as TC21–500 and TC21–600 in the subsequent discussion for simplicity. Subsequently, the TC21–600 samples were subjected to aging treatment. They were first heated to 830 °C for 30 min in a constant temperature box-type stove (KSL-1100X, Hefei Kejing Materials Technology Co., Ltd., Hefei, China) resistance furnace, followed by air cooling from 830 °C to 500 °C and holding for 60 min in the same furnace. Prior to aging, the samples were vacuum sealed in quartz tubes with argon gas to prevent oxidation during thermal treatment. This aging cycle was repeated four times, with air cooling to room temperature between each cycle. These specimens are referred to as TC21–600A. The specific heat-treatment process is shown in Figure 1.

### 2.2. Mechanical Properties

The hardness was measured using a nanoindenter (Hysitron Inc., Eden Prairie, MN, USA) with a trigonal diamond indenter, which was loaded to 3000 μN at a constant loading rate of 50 μN/s with a holding time of 5 s. The selected areas were marked with dots according to a matrix of 5 × 8, with a spacing of 5 μm between the neighboring dots. The testing process complies with GB/T 4340.1-2024 [27]. The dog-bone-shaped tensile specimens with a gauge length of 50 mm, a width of 10 mm, and a thickness of 1.5 mm were cut along the rolling direction (RD) by using wire electrical discharge machining. Room temperature uniaxial tensile experiments were conducted on an AG-X plus PC-controlled mechanical testing system (Shimadzu, Kyoto, Japan) with a constant strain rate of 1.0 × 10^−3^ s^−1^. To ensure the accuracy and repeatability of tensile data, three samples of each type were tested in parallel, and the average was calculated. The testing process complies with GB/T 228.1-2021 [28].

### 2.3. Microstructure Characterization

The phase identification of the alloy was performed using X-ray diffraction (XRD, SmartLab, Rigaku Corporation, Tokyo, Japan) using Cu-Kα radiation and a scanning 2θ range of 30–80 ° at a rate of 2 °/min. Before detection, the samples were mechanically ground and polished with diamond paste (d < 0.5 µm). Microstructural characterizations were carried out by using scanning electron microscopy (SEM, ULTRA55, Carl Zeiss Nanomaterials GmbH, Oberkochen, Germany) equipped with an electron backscatter diffraction detector (EBSD, Crossbeam 550, Carl Zeiss Nanomaterials, Oberkochen, Germany). The samples for SEM observations were polished and etched for 50 s in Kroll reagent consisting of 10% HF, 20% HNO_3_ and 70% H_2_O. Before EBSD analysis, the specimens underwent grinding and electro-polishing in a solution (6% perchloric acid, 34% n-butanol, and 60% methanol) with a working voltage of 27 V and a temperature of −15 °C for ~ 60 s. The EBSD with HKL Channel 5 software (Oxford Instruments) was employed for observation at a step size of 500 nm operating at an accelerating voltage of 20 kV. The data were analyzed using AZtec software (Oxford Instruments). The sample coordinates were defined as rolling direction (RD), transverse direction (TD), and normal direction (ND). The observed sections of the samples for EBSD were in the RD-TD plane. Specimens for TEM observations were ground to a thickness of 40–50 µm using sandpapers with various grades, and then discs with a diameter of 3 mm were cut. Subsequently, the discs were thinned by using electrolytic twinjet (Tenupol-5, Struers, Ballerup, Denmark) polishing at 28 V and 40 mA in a solution containing 6% perchloric acid, 34% n-butanol, and 60% methanol. The obtained foils were further ion milled via Gatan 695 precision ion polishing system (PIPS-695, Gatan, Pleasanton, CA, USA), and the observations were conducted on a transmission electron microscope (TEM, JEOL, JSM-2100F, Tokyo, Japan) at 200 kV.

## 3. Results

### 3.1. Microstructures

**Figure 2:**
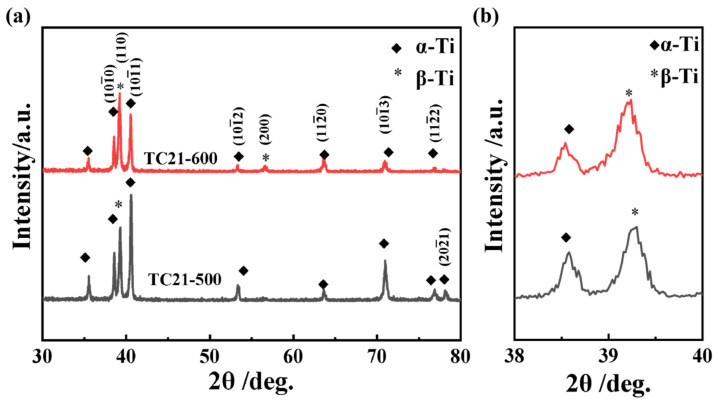
XRD diffraction patterns of TC21–500 and TC21–600 (**a**) and the magnified section of the peaks near 39° (**b**).

Figure 2 presents the XRD patterns of the TC21 titanium alloy after warm rolling at different temperatures, showing that the alloy primarily consists of α and β phases (Figure 2a). Evidently, these peaks correspond to both α and β phases. The α phase predominantly develops along crystallographic planes (10$\bar{1}$0), (10$\bar{1}$1), (10$\bar{1}$2), (11$\bar{2}$0), (10$\bar{1}$3), (11$\bar{2}$2) and (20$\bar{2}$1), while the β phase primarily forms along the (110) crystallographic plane. After rolling at 600 °C, the α-phase diffraction peaks (20$\bar{2}$1) nearly vanish, accompanied by an augmentation in the intensity of the β-phase diffraction peak (200) (Figure 2a). The magnified section near 39° clearly shows that the β-phase diffraction peaks shift toward lower angles at 600 °C (Figure 2b), which may be attributed to significant lattice distortions induced by severe deformation during rolling.

**Figure 3:**
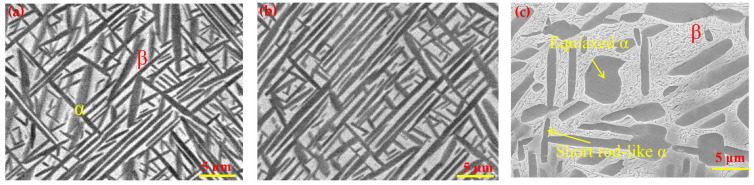
SEM images show the microstructure of TC21–500, TC21–600 and TC21–600A (**a**–**c**), respectively.

Figure 3a–c display the SEM microstructures of the sample rolled at 500 °C (TC21–500) and 600 °C (TC21–600). A common feature is that the microstructures of the alloys are primarily composed of lamellar α-phase and retained β laths (Figure 3a,b). The lamellar α phase in the dark is uniformly distributed within the β matrix in gray and exhibits directional growth. With increasing warm-rolling temperatures, the average thickness and spacing of the α lamellar significantly decrease. The average thickness of the α lamellar is 1.19 and 0.95 µm for TC21–500 and TC21–600, respectively, while the spacing is approximately 3 and 2 µm. After aging at 500 °C for 4 h (named as TC21–600A), the microstructure (Figure 3c) reveals a complex distribution consisting of equiaxed α grains, short rod-like α and the β matrix with irregular shape. Approximately 20% of the structure consists of equiaxed α phase, which formed by the spheroidization of the lamellar α phase during aging.

**Figure 4:**
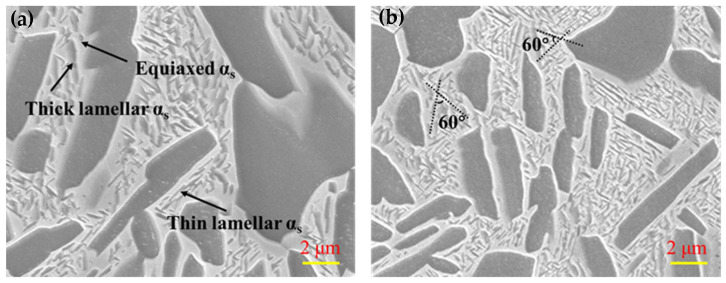
SEM images showing the distribution and morphologies of α_s_ phase in TC21–600A (**a**,**b**).

Figure 4a,b show the SEM microstructures of the α_s_ phase. Distinctly, the nanosized α_s_ phase is dispersedly located in the β phase, showing various morphologies such as equiaxed, thin and thick lamellar α_s_ phases. Among them, the α_s_ lamellae are dominant. As shown in Figure 4b, the neighboring α_s_ lamellae demonstrate a specific orientation relationship, characterized by a 60° angle between them. The triangular structure formed between adjacent α_s_ phases further divides the β-phase matrix into nanoscale blocky β phases. It has been reported that this structure is helpful for reducing the overall strain energy of the materials [29,30]. The formation of this specific microstructure is related to the selection of α_s_ variants. In titanium alloys, the relationship between β and α_s_ defers to a Burgers orientation relationship, namely (0001)α//(110)β, <11$\bar{2}$0>α//<111>β. There are multiple equivalent (110) planes and <111> directions within a β grain, so various α_s_ variants with different orientations can be generated during the phase-transformation process.

**Figure 5:**
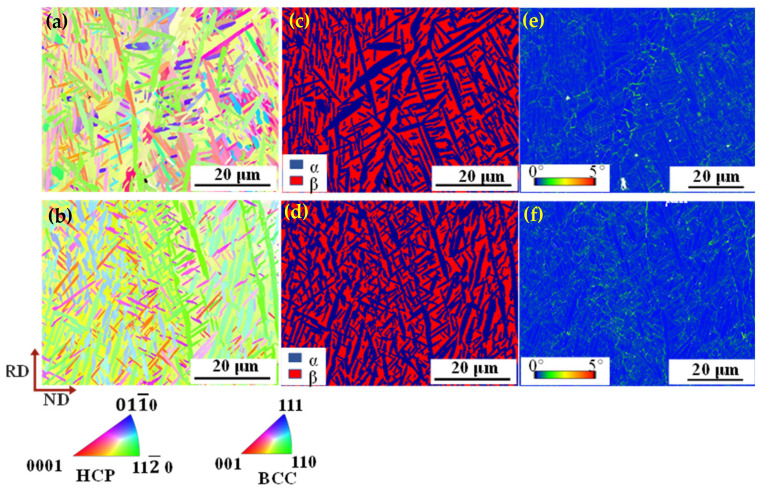
IPF (**a**,**b**), phase distribution (**c**,**d**) and KAM maps (**e**,**f**) for TC21–500 and TC21–600.

Figure 5 exhibits the inverse pole figure (IPF), phase distribution and kernel average misorientation (KAM) maps. As shown in the IPF maps (Figure 5a,b), a greater number of lamellar and equiaxed α phases are located in the β matrix of TC21–600 compared to TC21–500. It is worth noting that both the β and α phases demonstrate distinctly preferential orientations, which become more pronounced with the increase of rolling temperatures. As far as the β phase is concerned, the grains have a pronounced <110>β//RD (rolling direction) relationship, which is attributed to the formation of a fiber texture in the body-centered cubic (BCC) phase during rolling [31]. The α fiber texture in the BCC phase is characterized by <110> direction parallel to the rolling direction, i.e., <110>β//RD. Additionally, the α phase displays a <11$\bar{2}$0>α//RD orientation relationship, mainly due to the development of a prismatic and basal texture. Figure 5c,d display the corresponding phase images of TC-500 and TC-600. Distinctly, a greater number of thin α lamellae occur in the β matrix with the increase of rolling temperatures, demonstrating an interlocked structure. The KAM map for the sample rolled at 500 °C (Figure 5e) reveals a high density of stored geometrically necessary dislocations (GNDs) near the interfaces of α laths, and the value is estimated as the order of 3.22 × 10^14^ m^−2^, mainly because of the strength difference between α and β phases and the effective hindrance of dislocation slip by α/β interfaces. With increasing rolling temperature, a nucleation of the α phase takes place at the boundaries of the β phase, leading to an increase of α/β interfaces. This explains the higher GND density of 5.33 × 10^14^ m^−2^ near β-grain boundaries at 600 °C (Figure 5f).

**Figure 6:**
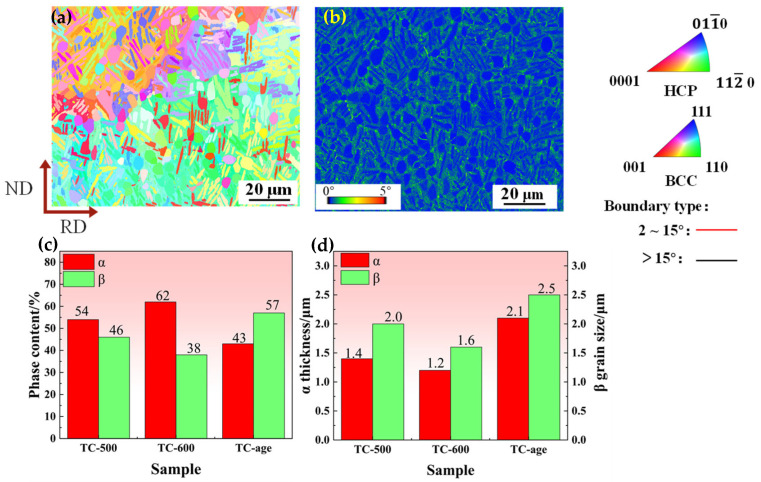
The IPF and KAM maps (**a**,**b**) of TC21–600A. The statistical plots of α- and β-phase contents and sizes (**c**,**d**).

TC21–600A consists of equiaxed and lamellar α in the β matrix (Figure 6a). Additionally, compared to the alloys before aging (Figure 5), the grain orientations become more random (Figure 6b). Very impressively, the intensities of <0001>α, <11$\bar{2}$0>α, <001>β and <110>β are extremely strong compared to those of <01$\bar{1}$0>α and <111>β. Among them, the special orientations including <11$\bar{2}$0>α and <110>β already existed before the aging process. After aging, no significant changes occur, i.e., misorientation is inherited by the newly formed grains. In addition, the aging process also induces the nucleation of β-phase grains possessing a predominant <001> orientation. Figure 6b shows the KAM map for TC21–600A. The aged sample demonstrates a higher dislocation density near the α/β interfaces, indicating dislocation pile-up and localized strain concentration at these regions. This is attributed to the spheroidization of lamellar structures during aging, which refines grains and increases the α/β interfaces. Regarding phase evolution across different types of processing, statistical analysis of the phase content and grain size are summarized in Figure 6c,d. The volume fraction of the α phase significantly increases from 54% to 62%, while the β phase decreases from 46% to 38% when the temperature rises from 500 °C to 600 °C. Concurrently, the thickness of the α phase is slightly refined from 1.4 μm to 1.2 μm. This result is attributed to the high temperature promoting spheroidization, generating fine α particles. In contrast, an obvious decrease in the volume fraction of the α phase is observed in TC21–600A. It indicates not only that the β→α_s_ transition occurred (Figure 3c), but also that a α→β transition happened and became predominant. This should be related to the occurrence of pseudo-spinodal decomposition during aging because of the elemental diffusion between α and β [32]. The metastable β_m_ phase undergoes decomposition from β_m_ → β + α_s_. However, the α-phase transforms to the β phase during the aging process because it is more stable at low temperature. After aging, the volume fraction of α drops from 62% to 43%, while the corresponding thickness of the lamellae increases from 1.2 µm to 2.1 µm (Figure 6). In contrast, the volume fraction of β elevates from 38% to 57%, and the grain size increases from 1.6 µm to 2.5 µm.

**Figure 7:**
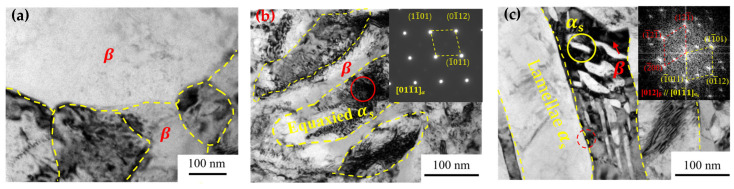
TEM images of TC21–600A before tensile testing. The morphologies of α_s_ and β phases, with an insect showing the SAED patterns of two phases (**a**–**c**).

To elucidate the strengthening mechanisms, TEM observations were performed to explore the microstructure (Figure 7). After aging, a few recrystallized grains of β with a size of ~ 100 nm occur at the vicinity of grain boundaries (Figure 7a), suggesting that recovery of dislocations took place during the aging process. Aside from the lamellar α_s_ phase, an equiaxed α_s_ phase can be observed. Obviously, the equiaxed α_s_ grains contain more dislocations than the lamellar α_s_; meanwhile, the β phase always has dense dislocations (Figure 7b,c). This demonstrates that the equiaxed α_s_ grains can deform compatibly with the β phase during warm rolling, which is beneficial for hindering the premature initiation and propagation of the cracks, thus enhancing the ductility. Conversely, the α_s_ lamellar effectively impedes dislocation motion (Figure 7c), thereby enhancing strength, which is consistent with the results from Gao et al. [20]. Meanwhile, within the β lamellae, numerous equiaxed nanoscale α_s_ phases (indicated by the yellow circle) are observed to be tangled with dense dislocations (Figure 7c). These nanosized α_s_ particles effectively hinder dislocation movement, thereby increasing the yield strength [33,34]. Selected area electron diffraction (SAED) and Fourier transform (FT) analysis were performed on the regions in red circles (Figure 7b,c), revealing that the α_s_ and β phases have HCP and BCC structures, aligning with an orientation relationship of [012] β//[01$\bar{1}$1] α_s_.

**Figure 8:**
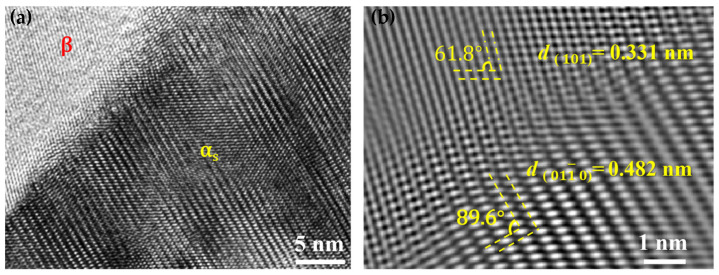
A HRTEM picture of TC21–600A (**a**) and the magnified view of the box region reveals the planar spacing of (101) β and (01$\bar{1}$0) α_s_ (**b**).

The crystal plane spacing of (101) is 0.331 nm and the angle between the two neighboring planes is 61.8° for the β phase; similarly, the planar spacing of (01$\bar{1}$0) is 0.482 nm, and the interplanar angle is 89.6° for the α_s_ phase (Figure 8a,b). The mismatch between the two phases at the interface is calculated by [35]:

(1) $$\delta =\frac{2\left(a_{\alpha }-a_{\beta }\right)}{a_{\alpha }+a_{\beta }}$$

where $a_{\alpha }$ and $a_{\beta }$ are the lattice constants of the α_s_ phase and β phase, respectively. The calculated δ with a value of 0.37, hence, the interface between α_s_ and β is semi-coherent, which makes dislocation slip difficult and causes extra strengthening.

### 3.2. Mechanical Properties

**Figure 9:**
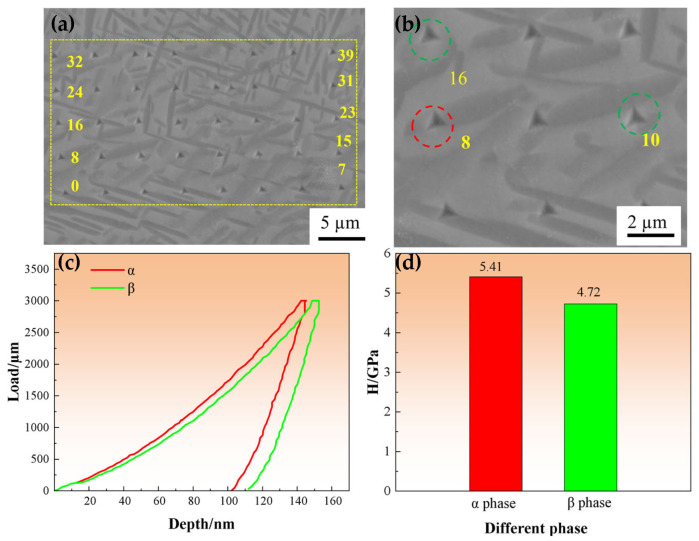
Nanoindentations of TC21–600A. A square and arrows showing the route and numbers of the nanoindentations (**a**), enlarged image exhibits the precisive location of the nanoindentations (**b**), typical loading–unloading curves of the α and β phases (**c**), and average hardness of the two phases (**d**).

The indentation morphology of nanoindentation was observed by SEM for distinguishing the hardness of duplex phases in TC21–600A (Figure 9a). Forty locations were selected for indentation testing, and the number and sequence of the indentations are indicated by the data and red arrows. The 8# indentation is precisely located in the interior of the α phase, while the 10# and 16# indentations indicate the β phase (Figure 9b). Indentations that fell on the interfaces were excluded in the analysis. Figure 9c shows the typical loading–unloading curves for the α and β phases; obviously, the α phase has a higher hardness. Statistical analysis revealed that the α phase exhibits an average hardness of 5.41 GPa, while the β phase demonstrates a lower hardness of 4.72 GPa (Figure 9d). The pronounced strengthening advantage of the α phase suggests its predominant role in bearing applied loading and governing the yield strength of TC21–600A. This discrepancy can be attributed to the crystallographic characteristics of the two phases: the hexagonal close-packed (HCP) structure of the α phase offers limited slip systems, consequently imposing greater resistance to dislocation motion and plastic deformation, whereas the body-centered cubic (BCC) structure of the β phase possesses more abundant slip systems, facilitating plastic flow at lower stress levels.

**Figure 10:**
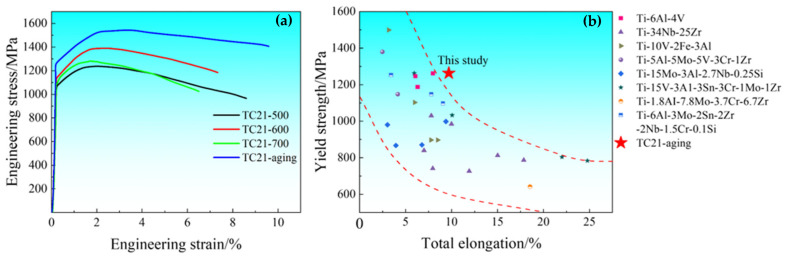
(**a**) Engineering stress–strain curves of TC21–500, TC21–600 and TC21–600A. (**b**) A comparison of σ_y_ and ε_f_ of TC21–600A with the reported Ti alloys: Ti-6Al-4V [36], Ti-34Nb-25Zr [37], Ti-10V-2Fe-3Al [38,39], Ti-5Al-5Mo-5V-3Cr-1Zr [40], Ti-15Mo-3Al-2.7Nb-0.25Si [41], Ti-15V-3A1-3Sn-3Cr-1Mo-1Zr [42,43], Ti-1.8AI-7.8Mo-3.7Cr-6.7Zr [44], Ti-6Al-3Mo-2Sn-2Zr-2Nb-1.5Cr-0.1Si [45].

Figure 10a shows the engineering stress–strain curves of the warm-rolled and aged TC21 alloys. TC21–500 exhibits a yield strength (σ_y_, yield strength at 0.2% offset) of ~1070 ± 10 MPa and an elongation-to-failure (ε_f_) of 8.6%. With increasing the warm-rolled temperature to 600 °C, the σ_y_ reaches a maximum of 1138 ± 7 MPa. In contrast, the ε_f_ shows an inverse relationship with the rolling temperatures, decreasing more noticeably from 8.6% at 500 °C to 7.3% at 600 °C. The sample rolled at 600 °C was aged at 500 °C, and the resultant sample (TC21–600A) demonstrates a significant improvement in mechanical properties. The σ_y_ increases by 125 MPa to 1263 MPa, and the ε_f_ increases by 2.3% to 9.6% compared to TC21–600. Notably, TC21–600A not only exhibits the highest σ_y_ but also maintains a superior strain-hardening rate throughout the plastic deformation stage compared to the warm-rolled specimens. This suggests a significantly enhanced strain-hardening ability to resist premature necking. Figure 10b presents a direct comparison of the σ_y_ and ε_f_ between TC21–600A in this study and other high-performance β-type and (α + β) titanium alloys reported in the literature, which include the aged and unaged conditions. Compared with β-type titanium alloys [36,37,38,39,40,41,42,43,44], TC21–600A exhibits a more excellent σ_y_ with only a slight loss in the ε_f_. In contrast, relative to other (α + β) titanium alloys such as Ti-6Al-3Mo-2Sn-2Zr-2Nb-1.5Cr-0.1Si [45], TC21–600A achieves a higher σ_y_ without compromising ductility. The comparative analysis reveals that TC21–600A demonstrates better comprehensive properties. 

## 4. Discussion

### 4.1. Deformation Mechanism

**Figure 11:**
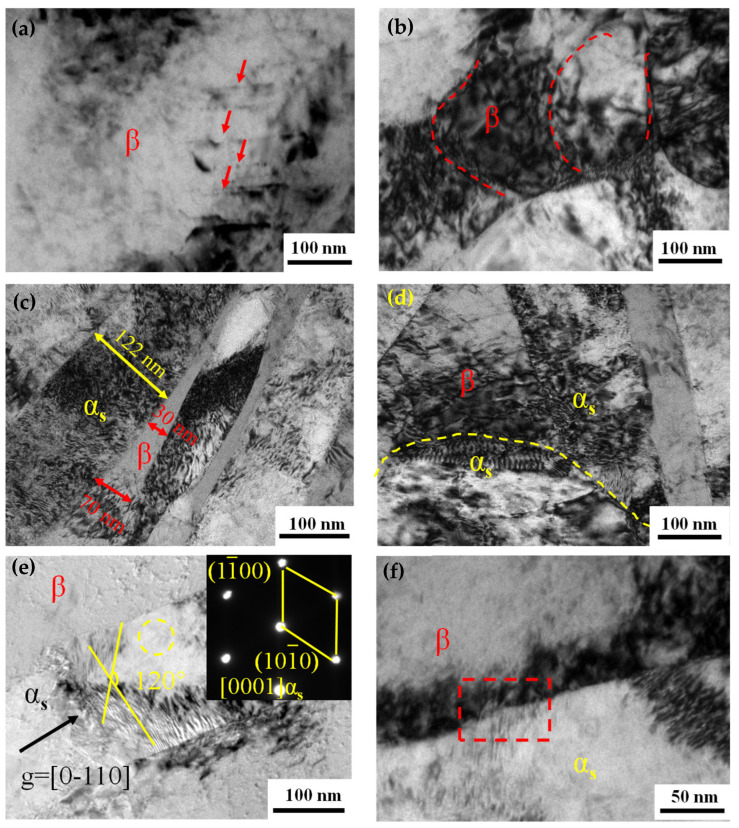
TEM images of TC21–600A after tensile testing. (**a**,**b**) β phase, (**c**–**e**) α_s_ lamellar structure, (**f**) the interface between α_s_ and β phases.

To investigate the deformation mechanism of the TC21–600A, TEM characterization was performed. Figure 11 shows TEM images of the fracture surfaces in the deformed TC21–600A. An array of parallel slip lines formed within the β phase of TC21–600A (Figure 11a). It was found that the β phase contained nanoscale subgrain structures, accompanied with a high density of dislocation tangles (Figure 11b). These indicate that the β phase has suffered severe deformation and the subgrain structures should be associated with the accumulation and rearrangement of dislocations. Meanwhile, the tangled dislocations serve as an effective obstacle to the dislocation slip, thereby strengthening the β phase. The deformation behavior of the β lamellar varies with thickness (Figure 11c); a fine size effectively suppresses the initiation and motion of dislocations. Thus, it is reasonable to notice that the thicker lamella (70 nm) exhibits a higher dislocation density than the thinner one (30 nm). In addition, dense dislocations can be observed in the lamellar α_s_ phases (Figure 11c). This phenomenon is significantly different from that shown in Figure 9c. Close observations of the interfacial region between the α_s_ phase and β phase distinctly show that substantial dislocations pile up at the α_s_/β interfaces (Figure 11d,e), thereby causing severe stress concentration. To observe the lamellar α_s_ phases under g = [0001], two sets of intersecting slips with an angle of 120° are seen, corresponding to (01$\bar{1}$0) and (1$\bar{1}$00) prismatic slip, respectively. This scene should be attributed to the fact that the β phase deforms first, generating dislocations that are hindered at the phase boundaries. This pile-up creates stress concentration; when the stress concentration reaches a critical level, dislocations will shear through the semi-coherent interface (Figure 11f), enabling coordinated deformation. Simultaneously, the generation of dislocations in the α phase effectively relieves the concentrated stress at the interface, preventing premature crack initiation, hence improving ductility. This suggests that the semi-coherent interface between α_s_ and β phases contributes to the strength, while retaining considerable ductility.

**Figure 12:**
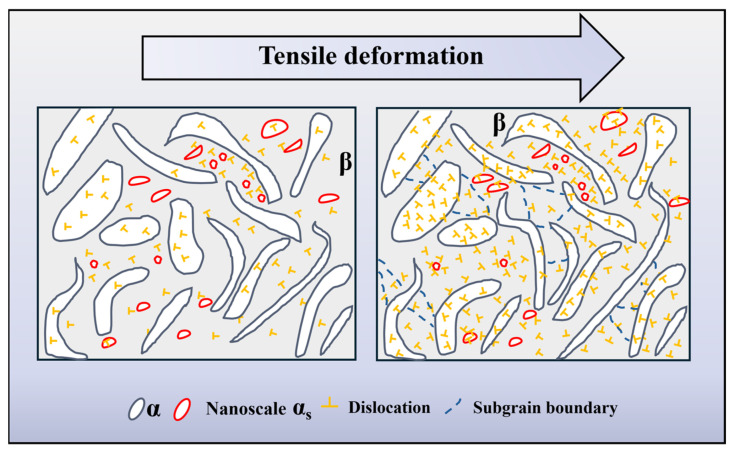
Schematic diagram revealing the deformation mechanisms of TC21–600A.

Based on the above analysis, the specific deformation mechanism of TC21–600A is shown Figure 12. During the aging process, spheroidization and fragmentation of the lamellar α phase occurs, accompanied by the precipitation of a high density of α_s_ phase in the β matrix. The soft β phase matrix undergoes plastic deformation first, generating GNDs that pile up at the α_s_/β interfaces. The fine lamellar and equiaxed α_s_ act as effective barriers to the dislocation movement due to their thin lamellar thickness and fine grain size, leading to the formation of dense dislocation tangles and the development of subgrain structures. This creates long-range back stress, which elevates the overall flow stress and contributes to the high and sustained strain-hardening rate. Obviously, the above observed heterostructure significantly enhances the strengthening effect. Furthermore, the presence of a semi-coherent α_s_/β interface allows the large lamellar and equiaxed α_s_ phase to synergically deform with the matrix once a critical stress is reached, thereby allowing dislocations to shear through the interface. As a result, the yield strength of the β phase increases while maintaining good ductility of TC21–600A.

### 4.2. Yield Strength

To clarify the origin of the high yield strength of TC21 titanium alloys in this study, the tensile yield strength is predicted by the following equation [46]:

(2) $$∆\sigma_{y}=∆\sigma_{gb}+∆\sigma_{S}+∆\sigma_{d}+∆\sigma_{\mathrm{Orowan}}$$

where $∆\sigma_{gb}$ is the grain boundary strengthening, $∆\sigma_{S}$ is solid solution strengthening, and $∆\sigma_{d}$ is dislocation strengthening. The grain boundary strengthening can be assessed through the rule of mixture, related to the combined effects of the lamellar thickness of α phase and the volume fraction of the β phase. According to the rule of mixtures, a calculation formula can be described as [47]:

(3) $$∆\sigma_{\mathrm{gb}}=\sigma_{\alpha }+f_{\beta }(\sigma_{\beta }-\sigma_{\alpha })+M_{\alpha }(1-f_{\beta })h^{-1/2}{+f}_{\beta }M_{\beta }{((1.2h\sqrt{f_{\beta }})/(1-\sqrt{f_{\beta }}))}^{-1/2\;}$$

where $\sigma_{\alpha }$ and $\sigma_{\beta }$ are the lattice friction of α and β phases, and h is the average thickness of the α lamellae. $M_{\alpha }$ and $M_{\beta }$ are the coefficients related to the chemical compositions and crystal structures. Here $\sigma_{\alpha }$, $\sigma_{\beta }$, $M_{\alpha }$ and $M_{\beta }$ are 945 MPa, 771 MPa, 7.11 MPa/mm^2^ and 12.76 MPa/mm^2^ [48], respectively. Consequently, the $∆\sigma_{\mathrm{gb}}$ can be calculated to be 850 MPa in TC21–600A by referring to Figure 6.

$∆\sigma_{s}$ related to the compositions of each element can be estimated by [49]:

(4) $$∆\sigma_{s}={\left(\sum_{i}B_{i}^{1.5}X_{i}\right)}^{2/3}$$

where $B_{i}$ and $X_{i\;}$ are the strengthening coefficient of the solute i and the atomic percentage, respectively. The specific values are listed in Table 2. Thus, $∆\sigma_{S}$ contributes 213 MPa to the yield strength.

The $∆\sigma_{d}$ is expressed as [50]:

(5) $$∆\sigma_{d}=\mathrm{MG}b\sqrt{\rho }$$

(6) ρ=2θ/μb

where M is a constant (0.56), G is the shear modulus value of 42 GPa and b is Burger’s vector value of 0.24 nm. *ρ* is the dislocation density, where θ is the average of KAM of all materials, and µ is the EBSD step size (0.5 nm). The $∆\sigma_{d}$ is calculated to be 122 MPa.

**Figure 13:**
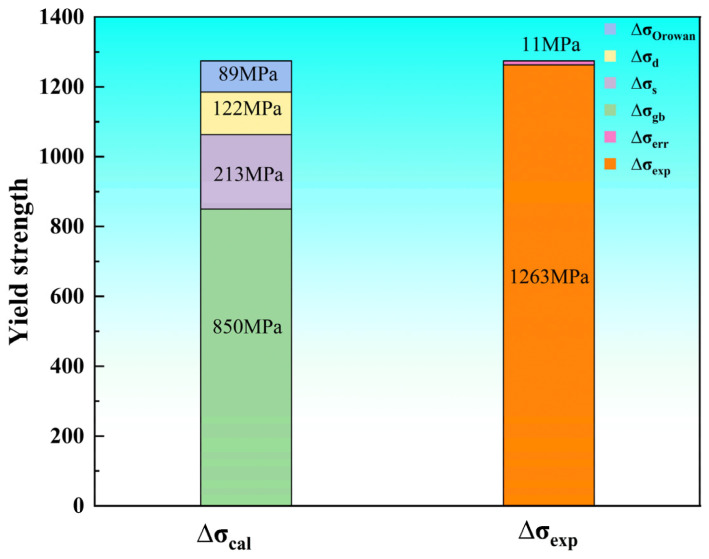
Contributions of various strengthening mechanisms to the yield strength of TC21–600A.

The $∆\sigma_{Orowan}$ can be quantified by the Ashby–Orowan equation [51]:

(7) $$∆\sigma_{O\mathrm{rowan}}=(0.583\mathrm{Gb}f^{1/2}/d)\ln\left(d/2b\right)$$

where *f* is the volume fraction of the α_s_ phase, and d is the average diameter of the α_s_ phase. It is noteworthy that the $∆\sigma_{O\mathrm{rowan}}$ only exerts a significant reinforcing effect on TC21–600A. This is because numerous fine α_s_ particles only exist in TC21–600A, resulting in a $∆\sigma_{O\mathrm{rowan}}$ of 89 MPa. The contributions of the various strengthening mechanisms to the overall yield strength are summarized in Figure 13.

Based on the calculations, it can be concluded that theoretical yield strength is well in agreement with the experimental tested value ($∆\sigma_{exp}$), with a negligible error ($∆\sigma_{err}$, ± 11 MPa), accounting for merely 1% of the total yield strength. It is noteworthy that the lamellar structures introduced during warm rolling are identified as the predominant strengthening factor, and the $∆\sigma_{gb}$ contributes over 67% of the overall strength. The second highest item is $∆\sigma_{s}$, accounting for 17% of the total yield strength.

### 4.3. Ductility

**Figure 14:**
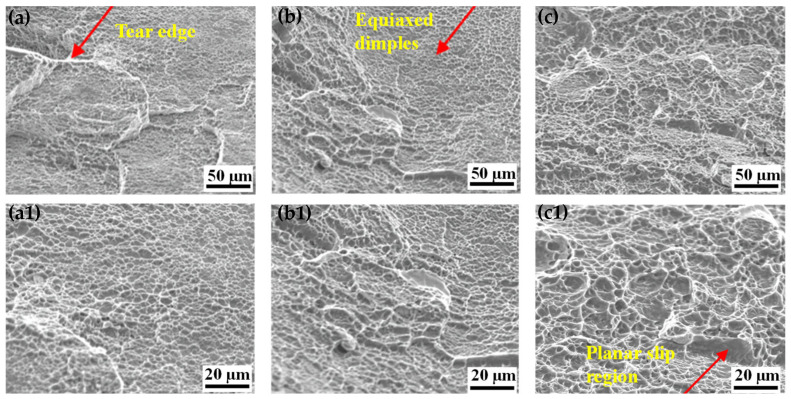
Fracture morphologies of TC21–500 (**a**,**a1**), TC21–600 (**b**,**b1**) and TC21–600A (**c**,**c1**).

The ductility of TC21–600 significantly enhances after aging, while maintaining an enhanced work-hardening rate. The elongation-to-failure increases from 7.3% to 9.6%, compared with the warm-rolled alloy, which should be related to the grain coarsening and dislocation recovery caused by aging. This is attributed to the reduction of the lamellar α-phase and texture intensity caused by the aging process. Figure 14 shows the fracture morphologies of TC21–500, TC21–600 and TC21–600A. The substantial tear ridges appear in the fracture surfaces of the warm-rolled alloys (Figure 14a,b1). This scene is mainly associated with the abundant lamellar α phase, which impedes dislocation motion and results in severe pile-ups at the interfaces between the α and β phases, ultimately leading to crack nucleation once the local stress concentration exceeds a critical threshold. Cracks grow along the α/β interfaces, thereby forming tear ridges. In TC21–600, the fracture surface shows mixed morphologies of dimples and cleavage-like facets, indicating a transition in fracture mode (Figure 14b,b1). The presence of deep dimples suggests improved micro-void coalescence compared to TC21–500 (Figure 14a,a1). In contrast, TC21–600A exhibits a fracture surface predominantly composed of equiaxed dimples with a few tear ridges. Evidently, the aging treatment induced the fragmentation of the lamellar α to an equiaxed morphology. This microstructural refinement allows dislocations to either shear through or bypass the α particles, thereby effectively enhancing the ductility of TC21–600A (Figure 14c,c1).

**Figure 15:**
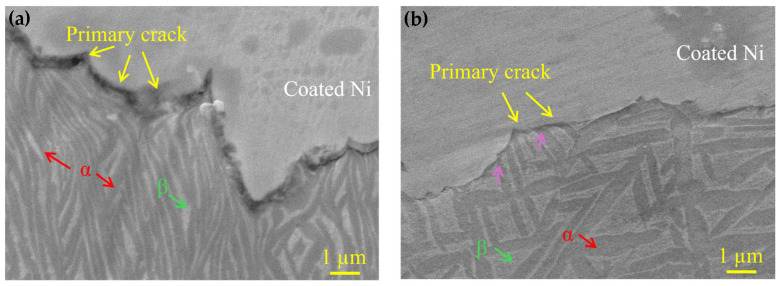
Typical SEM image of the region beneath the fracture surface showing a crack propagation path, (**a**) TC21–500, (**b**) TC21–600A.

To elucidate the influence of the lamellar α phase on crack initiation and propagation, microstructural characterization was performed on the longitudinal section of fractured specimens. Figure 15a shows the propagation path of the main crack in TC21–500, where the α and β phases are in grey and light, respectively. The fracture is predominantly intergranular, accompanied with a few transgranular fractures, which mainly propagate along the interfaces between α and β. This behavior mainly derives from the strong obstruction of the lamellar α phase to the crack propagation. The large-angle deflection observed in the main crack propagation path accounts for the alternating sequence of transgranular and intergranular fracture. The extensive tear ridges in TC21–500 are associated with this mechanism. In TC21–600A, the cracks exhibit a combination of transgranular and intergranular characteristics (Figure 15b). This change in crack propagation suggests an effective improvement in the ductility of TC21–600A, indicating an enhancement in fracture toughness [52]. This further demonstrates that the aging process effectively improved the mechanical properties of the TC21 alloy.

**Figure 16:**
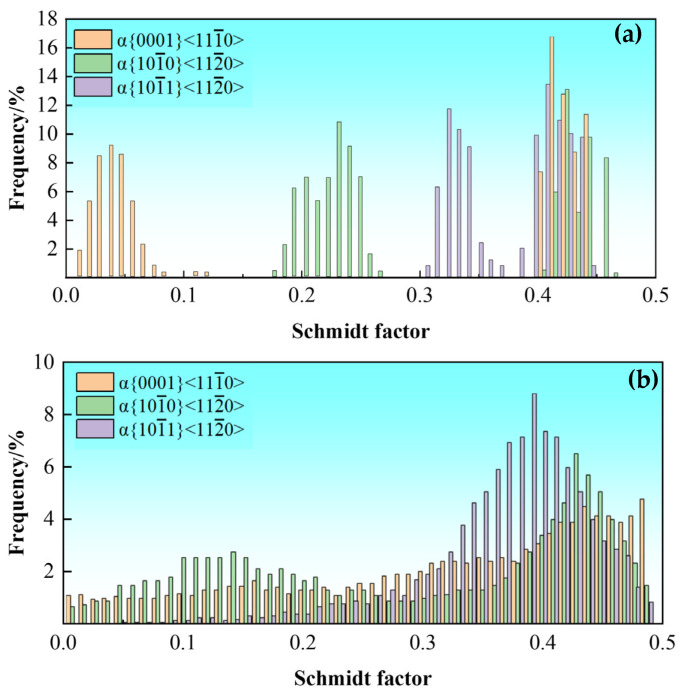
Distribution of Schmidt factors for α phase in TC21–600 (**a**) and TC21–600A (**b**).

The weakened crystallographic texture in the alloy is also conducive to the improved ductility. An excessively strong texture deteriorates the ductility of titanium alloys [53]. The IPF of TC21–600 confirms the existence of obvious prismatic and basal textures in the α phase (Figure 5b). In contrast, after aging treatment, the grain orientation became more random, and the strength of the prismatic and basal textures were significantly weakened (Figure 6b). In addition, the distribution of Schmid factors is used to determine the propensity for slip-system activation. Figure 16 shows the Schmidt factor distribution of the α phase before and after aging. There are five common slip modes for the α phase, namely the <a>-type basal slip {0001}<11$\bar{1}$0>, prismatic slip {10$\bar{1}$0}<11$\bar{2}$0>, pyramidal slip {10$\bar{1}$1}<11$\bar{2}$0>, Type I <c+a>-type pyramidal slip {10$\bar{1}$1}<11$\bar{2}$3> and Type II <c+a>-type pyramidal slip {11$\bar{2}$2}<11$\bar{2}$3>. Since the critical shear stress of the <a>-type slip system in the α phase is much lower than that of the <c + a>-type slip system, only the Schmidt factors under the <a>-type slip system (including basal, prismatic, and pyramidal slip) are counted. Prior to aging, the average Schmid factors of {0001} <11$\bar{1}$0>, {10$\bar{1}$0} <11$\bar{2}$0> and {10$\bar{1}$1} <11$\bar{2}$0> are 0.35, 0.35 and 0.38, respectively. After aging, the average Schmid factors of {10$\bar{1}$0} <11$\bar{2}$0> and {10$\bar{1}$1} <11$\bar{2}$0> increase to 0.36 and 0.39, respectively, thus making them more favorable for activation. This result is consistent with the good ductility of TC21–600A.

In summary, this study successfully constructed a heterogeneous structure composed of the micron β and nanoscale α_s_ as well as the equiaxed α phase in a TC21 alloy through a warm-rolling and aging treatment. As a result, excellent strength–ductility matching was achieved. Through the systematic analysis of the microstructure and strengthening mechanisms of TC21–600A, the cooperative effect of the nano-α_s_ phase, equiaxed α phase and β phase on the mechanical behavior of the alloy was revealed, which should shed light on the design of a lightweight alloy with ultrahigh strength that still retains good ductility.

## 5. Conclusions

This study comprehensively explored the mechanical properties and microstructural evolution of TC21 titanium alloy after warm rolling and aging at different temperatures by using SEM, EBSD, TEM, nanoindentation and tensile tests. The main conclusions can be drawn as follows:

(1) The TC21 titanium alloy warm rolled at 600 °C exhibits better combination of mechanical properties than at 500 °C, with a tensile strength of 1391 MPa, a yield strength of 1138 MPa, and a total elongation of 7.3%. Subsequent aging at 500 °C further enhances mechanical performance, reaching a yield strength of 1263 MPa, ultimate tensile strength of 1544 MPa and ductility of 9.6%.
(2) The sample aged at 500 °C has a microstructure composed of nanoscale α_s_ lamellae and micron-sized an equiaxed α_s_ phase, which cooperatively deform with the matrix; in contrast, the nanoscale α_s_ lamellae can effectively hinder dislocation motion, thereby providing a strengthening similar to the Hall–Petch type.
(3) The yield strength of the TC21 alloy is primarily attributed to the interface strengthening between α and β phases. After aging, the precipitation of nanoscale α_s_ lamellae in the interior of the β matrix provides additional strengthening, and the improved ductility can be related to the partial spheroidization of the lamellar α structure into an equiaxed morphology. Spheroidization reduces the stress concentration at the interfaces between the α and β phases and decreases the texture intensity of the α phase.

## Figures and Tables

**Table 1:** Chemical compositions of TC21 titanium alloy (mass percentage, mass%).

Al	Mo	Nb	Sn	Zr	Cr	Si	Fe	C	O	N	Ti
5.03	2.96	1.94	2.22	2.10	1.61	0.11	0.07	0.005	0.075	0.009	Bal.

**Table 2:** Mass and atomic percentage (at. %) and strengthening coefficient (B_i_) in TC21 alloys.

Elements	Mass%	at. %	B_i_ (MPa at. ^−2/3^)
Al	5.03	9.01	285
Mo	2.96	1.49	575
Sn	2.22	0.90	2303
Zr	2.10	1.11	1201
Nb	1.94	1.01	71
Cr	1.61	1.50	1665
Fe	0.07	0.06	1715

## Data Availability

All data used in this study are available upon request from the corresponding author.

## References

[1] Lütjering G. (1998). Influence of processing on microstructure and mechanical properties of (α+β) titanium alloys. Mater. Sci. Eng. A.

[2] He S.Y., Liang Y.L., Sun H. (2025). Design biomedical β-Ti alloys with exceptional strength-ductility balance via domain knowledge-based machine learning. Acta Mater..

[3] Chen L.M., Sun Q.Z., Xiao L.R. (2024). Effect of the subsolvus and supersolvus solution treatments on the basket-weave microstructure, room and high temperature properties of TC21 alloy. Mater. Sci. Eng. A.

[4] Luo H.Y., Zeng W.D., Chen H.W. (2025). Effect of microstructure on plastic deformation near the ASB, microcrack nucleation behavior and dynamic mechanical properties of TC21 alloy under dynamic compression. Mater. Sci. Eng. A.

[5] Ren L., Xia W., Kent D. (2020). Simultaneously enhanced strength and ductility in a metastable β-Ti alloy by stress-induced hierarchical twin structure. Scr. Mater..

[6] Patil U.S., Babu S.M.J., Thota M.K. (2025). Effect of heat treatment process parameters, cooling rate on microstructure morphology, mechanical behavior and texture evolution of two phase (α+β) Ti-6Al-4V alloy. J. Alloys Compd..

[7] Ji R., Zhu K., Zhang H. (2024). Microstructure evolution, mechanical response and strengthening models for TA15 titanium alloy during thermal processes: A brief review. J. Mater. Res. Technol..

[8] Boyer R.R. (1996). An overview on the use of titanium in the aerospace industry. Mater. Sci. Eng. A.

[9] Li L., Luo L., Yan J.J. (2015). Dynamic globularization and restoration mechanism of Ti–5Al–2Sn–2Zr–4Mo–4Cr alloy during isothermal compression. J. Alloys Compd..

[10] Seol J.B., Bae J.W., Kim J.G. (2020). Short-range order strengthening in boron-doped high-entropy alloys for cryogenic applications. Acta Mater..

[11] Hémery S., Villechaise P., Banerjee D. (2020). Microplasticity at room temperature in α/β titanium alloys. Metall. Mater. Trans. A.

[12] Zheng X., Zheng S., Wang J. (2019). Twinning and sequential kinking in lamellar Ti-6Al-4V alloy. Acta Mater..

[13] Zhang C., Zhang J., Bao X. (2024). Hierarchically ordered coherent interfaces-driven ultrahigh specific-strength and toughness in a nano-martensite titanium alloy. Acta Mater..

[14] Dumas O., Malet L., Hary B. (2021). Crystallography and reorientation mechanism upon deformation in the martensite of an α-α’Ti-6Al-4V dual-phase microstructure exhibiting high work-hardening rate. Acta Mater..

[15] Gao J., Huang Y., Guan D. (2018). Deformation mechanisms in a metastable beta titanium twinning induced plasticity alloy with high yield strength and high strain hardening rate. Acta Mater..

[16] Zherebtsov S., Murzinova M., Salishchev G. (2011). Spheroidization of the lamellar microstructure in Ti–6Al–4V alloy during warm deformation and annealing. Acta Mater..

[17] Chen Y., Wang K., Ren Z. (2024). Interaction between phase transformation and static recrystallization during annealing of rolled TC18 titanium alloy. J. Mater. Sci. Technol..

[18] Zhang S., Zhang Y., Zou Z. (2022). The microstructure and tensile properties of additively manufactured Ti–6Al–2Zr–1Mo–1V with a trimodal microstructure obtained by multiple annealing heat treatment. Mater. Sci. Eng. A.

[19] Shao H., Huang X., Ma Y. (2025). Quantitative investigation of the effects of basketweave microstructure on mechanical strength of α+β titanium alloy. J. Mater. Res. Technol..

[20] Gao S., Zhang M., Wang Z.X. (2025). Impact-resistant titanium alloy with fine equiaxed structure fabricated by powder metallurgy. J. Mater. Sci. Technol..

[21] Li P.B., Wang K., Chu S.Y. (2024). Stress-induced martensite transformation and mechanical properties of fine-grained Ti-10V-2Fe-3Al alloy fabricated by friction stir processing. Mater. Sci. Eng. A.

[22] Zhang Z., Jun T.S., Britton T.B. (2016). Determination of Ti-6242 α and β slip properties using micro-pillar test and computational crystal plasticity. J. Mech. Phys. Solids.

[23] Dong R., Li J., Kou H. (2018). Precipitation behavior of α phase during aging treatment in a β-quenched Ti-7333. Mater. Charact..

[24] Wu S.W., Wang G., Wang Q. (2019). Enhancement of strength-ductility trade-off in a high-entropy alloy through a heterogeneous structure. Acta Mater..

[25] Wu X.F., Lu Y., Wang M.J. (2025). Comparative study the effect of β-phase ratio on mechanical properties of Ti-4Al-2.5 V-1.5 Fe and Ti-6Al-4V alloys by heavy warm rolling followed by annealing. J. Alloys Compd..

[26] (2016). Designation and Composition of Titanium and Titanium Alloys.

[27] (2024). Metallic Materials—Vickers Hardness Test—Part 1: Test Method.

[28] (2021). Metallic Materials—Tensile Testing—Part 1: Method of Test at Room Temperature.

[29] Wang S.C., Aindow M., Starink M.J. (2003). Effect of self-accommodation on α/α boundary populations in pure titanium. Acta Mater..

[30] Dai J., Tang B., Wang C. (2025). Simultaneously achieving exceptional and heat treatment insensitive strength-ductility synergy in an α+β titanium alloy via tailoring silicide and heterogeneous α precipitates. J. Mater. Sci. Technol..

[31] Kestens L.A.I., Pirgazi H. (2016). Texture formation in metal alloys with cubic crystal structures. Mater. Sci. Technol..

[32] Hao M., Li P., Li X. (2022). Heterogeneous precipitate microstructure in titanium alloys for simultaneous improvement of strength and ductility. J. Mater. Sci. Technol..

[33] Zhang C.L., Bao X.Y., Zhang D.D. (2021). Achieving superior strength-ductility balance in a novel heterostructured strong metastable β-Ti alloy. Int. J. Plast..

[34] Lei L., Zhao Q., Zhao Y. (2021). Study on the intrinsic factors determining impact toughness of TC21 alloy. Mater. Charact..

[35] He C.W., Shen T.F., Xue W.Y. (2024). Nanosized κ-Carbide and B2 Boosting Strength Without Sacrificing Ductility in a Low-Density Fe-32Mn-11Al Steel. Nanomaterials.

[36] Markovsky P.E., Semiatin S.L. (2011). Tailoring of microstructure and mechanical properties of Ti–6Al–4V with local rapid (induction) heat treatment. Mater. Sci. Eng. A.

[37] Ozan S., Lin J., Li Y. (2015). Development of Ti–Nb–Zr alloys with high elastic admissible strain for temporary orthopedic devices. Acta Biomater..

[38] Srinivasu G., Natraj Y., Bhattacharjee A., Nandy T.K., Rao G.N. (2013). Tensile and fracture toughness of high strength β Titanium alloy, Ti–10V–2Fe–3Al, as a function of rolling and solution treatment temperatures. Mater. Des..

[39] Nyakana S.L., Fanning J.C., Boyer R.R. (2005). Quick reference guide for β titanium alloys in the 00s. J. Mater. Eng. Perform..

[40] Wu D., Hao M., Zhang T. (2023). Heterostructures enhance simultaneously strength and ductility of a commercial titanium alloy. Acta Mater..

[41] Du Z., He Q., Chen R. (2022). Rolling reduction-dependent deformation mechanisms and tensile properties in a β titanium alloy. J. Mater. Sci. Technol..

[42] Naydenkin E.V., Mishin I.P., Zabudchenko O.V. (2023). Structural-phase state and mechanical properties of β titanium alloy produced by rotary swaging with subsequent aging. J. Alloys Compd..

[43] Mishin I.P., Naydenkin E.V., Zabudchenko O.V. (2021). Evolution of structure, mechanical properties and fracture of β titanium alloy in the process of wire obtaining. Mater. Lett..

[44] Tian Y., Zhang B., Chen R. (2025). Shear band induced nano-equiaxed (α+ β) microstructure evolution and the associated synergistic strengthening mechanism in Ti-7Mo-3Cr-3Nb-3Al alloy. J. Alloys Compd..

[45] Zhang L., Wen Y., Liu Y. (2023). Cr-promoted formation of B2+ L21 composite nanoprecipitates and enhanced mechanical properties in ferritic alloy. Acta Mater..

[46] Li G.Q., Shen Y.F., Jia N. (2022). Microstructural evolution and mechanical properties of a micro-alloyed low-density δ-TRIP steel. Mater. Sci. Eng. A.

[47] Zhao Q., Sun Q., Xin S. (2022). High-strength titanium alloys for aerospace engineering applications: A review on melting-forging process. Mater. Sci. Eng. A.

[48] Sun J., Lu H., Zhang H. (2024). Effect of thermal exposure on microstructure and mechanical properties of Ti65 high-temperature titanium alloy deposited by laser direct energy deposition. Mater. Sci. Eng. A.

[49] Zhao G.H., Liang X.Z., Kim B. (2019). Modelling strengthening mechanisms in beta-type Ti alloys. Mater. Sci. Eng. A.

[50] Yin T.W., Shen Y.F., Jia N. (2025). Roles of multicomponent nanoprecipitates in strengthening and low-temperature fracture toughness of weld heat-affected zones in a HSLC steel. Int. J. Miner. Metall. Mater..

[51] Zhang J.J., Shen Y.F., Jia N. (2026). Multiscale heterostructure and grain rotation promote the coordinated deformation of a multi-principal element alloy. J. Mater. Sci. Technol..

[52] Shen Y.F., Xue W.Y., Liu Z.Y. (2010). Nanoscratching deformation and fracture toughness of electroless Ni–P coatings. Surf. Coat. Technol..

[53] Zhang J., Bermingham M.J., Otte J. (2024). Ultrauniform, strong, and ductile 3D-printed titanium alloy through bifunctional alloy design. Science.

